# Short‐range multispectral imaging is an inexpensive, fast, and accurate approach to estimate biodiversity in a temperate calcareous grassland

**DOI:** 10.1002/ece3.9623

**Published:** 2022-12-14

**Authors:** John Jackson, Clare S. Lawson, Celestine Adelmant, Evie Huhtala, Philip Fernandes, Rose Hodgson, Hannah King, Lucy Williamson, Kadmiel Maseyk, Nick Hawes, Andrew Hector, Rob Salguero‐Gómez

**Affiliations:** ^1^ Department of Biosciences University of Sheffield Sheffield UK; ^2^ School of Environment, Earth & Ecosystem Sciences The Open University Milton Keynes UK; ^3^ Department of Biology University of Oxford Oxford UK; ^4^ Department of Engineering Science, Oxford Robotics Institute University of Oxford Oxford UK; ^5^ Max Planck Institute for Demographic Research Rostock Germany

**Keywords:** autonomous monitoring, biodiversity drone, remote sensing, unmanned aerial vehicle (UAV)

## Abstract

Image sensing technologies are rapidly increasing the cost‐effectiveness of biodiversity monitoring efforts. Species differences in the reflectance of electromagnetic radiation can be used as a surrogate estimate plant biodiversity using multispectral image data. However, these efforts are often hampered by logistical difficulties in broad‐scale implementation. Here, we investigate the utility of multispectral imaging technology from commercially available unmanned aerial vehicles (UAVs, or drones) in estimating biodiversity metrics at a fine spatial resolution (0.1–0.5 cm pixel resolution) in a temperate calcareous grassland in Oxfordshire, UK. We calculate a suite of moments (coefficient of variation, standard deviation, skewness, and kurtosis) for the distribution of radiance from multispectral images at five wavelength bands (Blue 450 ± 16 nm; Green 560 ± 16 nm; Red 650 ± 16 nm; Red Edge 730 ± 16 nm; Near Infrared 840 ± 16 nm) and test their effectiveness at estimating ground‐truthed biodiversity metrics from in situ botanical surveys for 37–1 × 1 m quadrats. We find positive associations between the average coefficient of variation in spectral radiance and both the Shannon–Weiner and Simpson's biodiversity indices. Furthermore, the average coefficient of variation in spectral radiance is consistent and highly repeatable across sampling days and recording heights. Positive associations with biodiversity indices hold irrespective of the image recording height (2–8 m), but we report reductions in estimates of spectral diversity with increases to UAV recording height. UAV imaging reduced sampling time by a factor of 16 relative to in situ botanical surveys. We demonstrate the utility of multispectral radiance moments as an indicator of biodiversity in this temperate calcareous grassland at a fine spatial resolution using a widely available UAV monitoring system with a coarse spectral resolution. The use of UAV technology with multispectral sensors has far‐reaching potential to provide cost‐effective and high‐resolution monitoring of biodiversity.

## Graphical abstract

We demonstrate the utility of low‐cost, coarse, multispectral imaging from commercially available drones in characterising the biodiversity of a temperate calcareous grassland at a high spatial resolution.

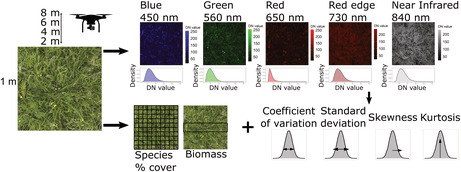

## 1 INTRODUCTION

With over one million species expected to go extinct by 2100, cost‐effectively monitoring biodiversity is a critical task in the Anthropocene (Díaz et al., 2019; Palmer et al., 2002). Image sensing technologies, which can be used to monitor biological systems through the measurement of reflected and emitted radiation, have emerged as a critical tool that can increase this cost‐effectiveness (Cavender‐Bares et al., 2022; Turner, 2014). The characterization of floral biodiversity with remote sensing, particularly with satellite imagery, is well‐established in biodiversity research (Pettorelli et al., 2005). Multiple efforts have been made toward using remote sensing data, particularly at large spatial scales and in forest ecosystems, to estimate plant diversity (Jetz et al., 2016; Tuanmu & Jetz, 2015; Turner et al., 2003). However, there are limitations in the use of long‐range remote sensing, including coarse spatial resolution that does not necessarily highlight biodiversity at small spatial scales (Gamon et al., 2020; Mairota et al., 2015), high sensor costs (e.g., hyperspectral sensor cost of $98,700, Headwall Photonics, 2022) and monitoring costs (e.g., flight cost of $60,000, Jet Propulsion Laboratory, 2022), and reliance on publicly available satellite data (e.g., The European Space Agency, 2022). Flexible application of remote sensing concepts and technology at a wide range of spatial scales, in variable environments, and with increased cost‐effectiveness, will provide vital resources for monitoring biodiversity (Cavender‐Bares et al., 2022; Turner, 2014).

Reflectance of electromagnetic (EM) radiation both including and outside the visible range (380–700 nm) has recently been demonstrated as an accurate proxy for biodiversity (Cavender‐Bares et al., 2020; Fassnacht et al., 2022; Wang & Gamon, 2019). Remotely sensed proxies for biological activity and biodiversity have been available for decades, including the normalized difference vegetation index (NDVI; Rouse et al., 1974), and there are now many spectral indices used in monitoring, including variance, entropy, and distance measures (Wang & Gamon, 2019). The general concept of “spectral diversity” is founded on the principle that, due to differences in functional form (both growth form and pigmentation), plant species have differential reflectance signals across the electromagnetic (EM) spectrum (Gamon et al., 1997). Thus, for a multispectral image, the diversity of spectral reflectance can be a proxy for the number of different plant species, or species diversity, when applied for an appropriate context and spatial scale (Fassnacht et al., 2022; Gholizadeh et al., 2019; Laliberté et al., 2020). Spatial scale, varying through the pixel resolution of multispectral image data, is central to spectral diversity's role as a proxy for biodiversity (Gamon et al., 2020; Wang et al., 2018). In prairie grassland ecosystems, associations between spectral coefficient of variation and biodiversity were only consistent for pixel resolutions below 5 cm, but this association varies depending on study site and the size of study organisms (Gamon et al., 2020). The spectral diversity concept was recently applied in the hyper‐diverse Cape Floristic Region, where destructively sampled leaf reflectance spectra were used to obtain a robust proxy ($R^{2}$ > .9) of species diversity across 1267–10 × 5 m quadrats (Frye et al., 2021). Therefore, integrating sensing data at a range of spatial scales (Laliberté et al., 2020; Turner, 2014) and the use of spectral surrogates for biodiversity for an appropriate biological context (Fassnacht et al., 2022) can rapidly improve biodiversity monitoring.

Recently, there have been several applications of spectral diversity from high‐resolution imaging data in grasslands (Conti et al., 2021; Gholizadeh et al., 2019; Lopatin et al., 2017). In prairie grassland ecosystems, close associations have been found between species diversity and spectral diversity, captured using aircraft‐mounted hyperspectral sensors and images at a spatial resolution of 1 × 1 m (pixel resolution; Gholizadeh et al., 2018, 2019, 2020). Gholizadeh et al. (2019) primarily use the average coefficient of variation across pixels and spectral bands as the metric of spectral diversity, which we also adopt here as a spectral distribution metric that is not dependent on mean reflectance. The association between spectral diversity and biodiversity has also since been demonstrated at a spatial resolution of 10 × 10 cm in coastal meadow habitats, but in a temperate meadow at a resolution of 3 cm a negative association with biodiversity was mediated by vertical complexity (Conti et al., 2021; Villoslada et al., 2020). Furthermore, at a fine resolution of <0.5 × 0.5 cm, static monitoring (i.e. sensor mounted to a fixed structure) of grassland plots has been used to estimate not only biodiversity metrics (Imran et al., 2021; Wang et al., 2018), but to reconstruct species percentage cover and extract detailed features of community dynamics (Lopatin et al., 2017). However, a key limitation of these close‐range imaging approaches is their reliance on expensive hyperspectral sensors (>$50,000 sensors; Gholizadeh et al., 2019; Imran et al., 2021; Lopatin et al., 2017) and monitoring ($1200 per hour using the CALMIT aerial sensor from Gholizadeh et al., 2019). Furthermore, previous studies have been focused in highly accessible, well‐studied areas for which precise image calibration (e.g., against solar interference) is more feasible, but image calibration may not be feasible in many field systems. Overcoming these cost and practical limitations will facilitate further use of spectral imaging in grassland biodiversity research.

Despite advances in image sensing, there is a need for cost‐effective and user‐friendly monitoring systems that are deployable at a fine spatial resolution. One potential solution is the use of commercial unmanned aerial vehicles (UAVs or drones), which have rapidly increased in popularity and off‐the‐counter availability over the last decade (Colomina & Molina, 2014). Here, we investigate the efficacy of coarse multispectral imaging from UAV technology in the estimation of biodiversity at a fine spatial resolution (0.1–0.5 cm pixel resolution) in a temperate calcareous grassland. We use a commercially available UAV system with a five‐band multispectral sensor (Blue 450 ± 16 nm; Green 560 ± 16 nm; Red 650 ± 16 nm; Red Edge 730 ± 16 nm; Near Infrared 840 ± 16 nm) to image 37–1 × 1 m quadrats that were also characterized using in situ biodiversity assessments from botanical surveys. Then, we use an analytical approach for which we do not calibrate raw at‐sensor radiance values to reflectance, which is not always practical or possible in field settings. Instead, we extract distribution metrics, which capture relative differences in at‐sensor radiance values within an image and estimate the repeatability of distribution metrics across the sampling period. Finally, we explore the association between these proxies of spectral diversity and biodiversity in this grassland community.

## 2 METHODS

### 2.1 Study site and in situ biodiversity data

Data collection took place at the two‐hectare section of the Upper Seeds field site (51°46′16.8″N 1°19′59.1″W; 165 m a.s.l) in Wytham woods, Oxfordshire, UK between 16th June and 14th July 2021, which is the peak of the growing season. The Upper Seeds site is a recovering and managed calcareous grassland, which was used for agriculture in the 1950s, before encroaching scrub vegetation was removed and the site was managed as a grassland beginning in 1978 (Gibson, 1986). Management on Upper Seeds is implemented with mowing of the site in mid‐July at the peak of the growing season, and again in early October, coinciding with the end of the growing season. The site has a low average soil depth (300–500 mm), generally alkaline soils (Gibson & Brown, 1991), a daily average temperature range of −5 to 26°C (2016–2020), a daily total precipitation range of 0–40 mm (2016–2020), and high general biodiversity, in which graminoids are the dominant functional group (59.1% by biomass). A total of 37 1 × 1 m experimental quadrats were used in the current study, which displays a large degree of variation in species composition and biomass. There were between 16 and 33 vascular plant species per m^2^, with a mean richness of 25.77 species and a median richness of 26 species. Total above‐ground dry biomass across quadrats varied between 166.8 and 931.5 g/m^2^, with a mean of 397.9 g/m^2^ and median of 327.2 g/m^2^. For the same sampling period, the community average (community weighted mean for most abundant species) plant height in control plots was 43.3 cm, and the community average specific leaf area (per mg of dry mass) was 0.23 cm^2^/mg.

We explored biodiversity and spectral diversity associations in the context of two long‐term experiments that aim to explore the response of grasslands to environmental change (full site map in Figure S1). These experiments are the Disturbance and Resources Across Global Grasslands (DRAGNet, *n* = 20 plots) coordinated research network (https://nutnet.org/dragnet) and the global drought network (DroughtNet, *n* = 17 plots) coordinated research network (https://drought‐net.colostate.edu/). All DRAGNet plots (5 × 5 m plots) were ambient controls, with no experimental treatments applied prior to the collection of the data reported here. Each 5 × 5 m plot from DroughtNet was one of four experimental treatments: ambient control plots (*n* = 5), −50% rainfall shelters to simulate drought (*n* = 5), +50% irrigated plots to simulate increased rainfall (*n* = 5), and procedural controls (rainfall shelter with no change to rainfall; *n* = 2; three plots were inaccessible for the UAV as the rainfall shelters were fixed). For analyses, ambient control treatments (*n* = 25) across the two research networks were pooled as we did not observe substantial differences between biodiversity metrics (Figure S2). To account for replicated observations of the same quadrats and estimate the consistency of spectral diversity measures, we explored quadrat‐level variance using mixed‐effects models with random effects for the quadrat ID.

**FIGURE 1:**
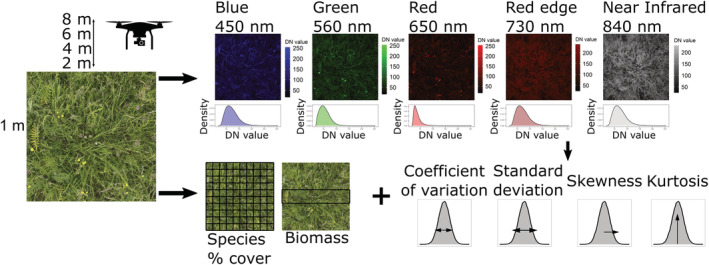
Schematic for assessing the efficacy of spectral distribution moments for capturing biodiversity in a temperate calcareous grassland. For each 1 × 1 m observation quadrat, we collected both UAV image data (top) and in situ biodiversity data (bottom). In situ biodiversity data were collected by botanical surveys for vascular plant percentage cover across the quadrat (from which richness, Shannon–Weiner, and Simpson's indices were calculated) and using dry above‐ground biomass for clip strips (area determined by the coordinated research networks DRAGNet and DroughtNet; see 2.1), after UAV images were taken. UAV images were collected for each plot at four recording heights (2, 4, 6, and 8 m) across five multispectral bands for which at‐sensor radiance digital number (DN) value distributions were summarized using four moments. Finally, in situ biodiversity data were compared with spectral distribution moments to examine their potential relationships using a Bayesian linear regression framework.

To estimate the efficacy of multispectral sensors in predicting biodiversity, we collected data from two sources, in situ biodiversity assessments and UAV‐derived multi‐spectral image data (Figure 1). For the in situ assessments, we quantified biodiversity metrics using species‐level percentage cover and dry above‐ground biomass data. We estimated percentage cover data for all vascular plant species in a plot using a 1 × 1 m gridded quadrat (10 cm grid), focusing on four broad functional groups: graminoids, legumes, forbs, and woody species (Figure 1). Because species overlapped spatially, percentage cover estimates could exceed 100%. Using relative proportions, p, calculated from percentage cover estimates, we calculated three biodiversity metrics: (i) vascular plant species richness, (ii) the Shannon–Weiner diversity index, H (Equation 1; Shannon & Weaver, 1963), and (iii) the Simpson's diversity index, D (Equation 2; Simpson, 1949):

(1) H=−∑plnp,

(2) $$D=\sum p^{2}$$

We estimated above‐ground biomass after UAV image/percentage cover data collection, using a clip strip of all vascular plant material in 1 × 0.2 m (DRAGNet; collected from standardized locations in the plot) or 1 × 0.25 m (DroughtNet; collected from the centre of each quadrat). Clip strips were gathered using hand trimmers at a height of 1‐2 cm above the soil surface. Within 1 day of collection, we sorted clip strips in to five functional groups: graminoids, legumes, forbs, woody species, and bryophytes (not included in species‐level percentage cover estimates) and dried them at 70°C for 48 h, before weighing the dry biomass at an accuracy of ±0.1 g. The estimates of biomass were scaled to g/m^2^ for analyses.

### 2.2 UAV image data collection

To obtain spectral diversity data, we collected image data using manual flights of the DJI Phantom 4 multispectral UAV (https://www.dji.com/p4‐multispectral). The sensor payload of the DJI Phantom 4 multispectral consists of six 4.96 × 3.72 mm complementary metal–oxide–semiconductor (CMOS) sensors: one RGB sensor for visible range color images, and five monochrome sensors for multispectral imaging. The five multispectral sensors are sensitive at the following electromagnetic wavelengths: Blue – 450 ± 16 nm, Green – 560 ± 16 nm, Red – 650 ± 16 nm, Red edge – 730 ± 16 nm, and Near‐infrared – 840 ± 26 nm (Figure 1). Each sensor has an effective resolution of 2.08 MP. All six image sensors are triggered simultaneously when capturing data, with negligible (but non‐0) positional differences between sensors. A dorsal spectral sunlight sensor on the P4 multispectral sensor provides image exposure compensation of multispectral image data, partially accounting for differences in solar radiation between images and ensuring radiance values were more comparable between images.

To obtain spectral diversity metrics, we collected multispectral images for each quadrat over several flights across the sampling period, capturing quadrat‐level variability with weather/light conditions. However, to minimize visual interference (from rain or low sun), all images were taken during dry weather and between 10:30 and 15:30 (BST). The corners of each DRAGNet quadrat were marked with flags (Figure S1b). For DroughtNet, the quadrat was approximated using the outer edges of the 5 × 5 m plot. All images were collected facing the western edge of each plot. We collected images at increasing approximate image recording heights of 2, 4, 6, and 8 m above the ground to capture changes in image resolution and consequences for estimating biodiversity (Figure 1). Flying height is recorded relative to the UAV's take‐off location, and although the topographical variation at the site is <5 m, image record heights were approximated using structures of known height (i.e., rainfall shelters, see Figure S5). Therefore, using these approximate image recording heights and sampling quadrats, the approximate pixel resolution of multispectral images was between 0.1 and 0.5 cm. Given community averaged heights of <50 cm and specific leaf area of 0.23 cm^2^/mg, pixel sizes of 0.1–0.5 cm were appropriate to distinguish between plant structures both within and between species. A total of 1878 individual images were collected for the 37 quadrats over seven sampling days.

### 2.3 Image processing

To extract spectral reflectance metrics from the raw image data, we standardized raw images across quadrats for each sample. The raw images encompassed the full field of view of the sensors, and we first batch‐cropped images with Adobe Lightroom v. 5 (Adobe, 2021) to include only data for the desired 1 m^2^ quadrats using flags as identification tools (where possible). Images were exported as .tif files maintaining at‐sensor radiance values with minimal post‐processing.

In contrast to other studies, we did not perform post‐processing to account for additional interference from solar radiation by normalizing at‐sensor radiance Digital Number (DN) values to reflectance values (Conti et al., 2021; Gholizadeh et al., 2019; Schläpfer et al., 2020). Solar radiation, which varies across the day and with weather conditions, influences radiance detected by image sensors. Therefore, typically objects with known reflectance are used as a reference to calibrate multispectral images from at‐sensor radiance to reflectance values using linear transformations (e.g., Conti et al., 2021). Therefore, the noise introduced from solar radiation means that DN values are not directly comparable between images. However, many instances may arise where calibration with known reflectance is not practical or possible in synchrony with image recording. These instances include the inaccessibility of sampling sites from the landing/calibration area (with differing light conditions) and changes in weather and radiation within a flight. Therefore, we opted to use an alternate approach that does not use calibrated reflectance values. First, we used only distribution metrics of the at‐sensor radiance values (Gholizadeh et al., 2019), particularly the spectral coefficient of variation, a variance metric that is corrected by the mean in each sample. Although mean radiance values are not directly comparable between images, relative differences between radiance values, that is, their distributions, are consistent. Second, we collected several repeated images of each quadrat across sampling days, which varied in weather conditions and levels of solar radiation. Then, we explicitly modeled the additional noise introduced by solar radiation using mixed‐effects models, with quadrat as a random effect and the quadrat‐level variance captured with repeatability analyses. Therefore, distribution metrics from radiance values repeated over sampling events should capture overall patterns in spectral diversity. Multispectral .tif images were treated as rasters for further image processing, and all subsequent analysis was carried out using R version 4.0.5 (R Core Team, 2021).

We calculated moments of spectral radiance for each image using the *raster* package (Hijmans, 2020). Following Gholizadeh et al. (2019), we calculated the coefficient of variation, standard deviation, skewness, and kurtosis across raster pixels to capture the shape of the at‐sensor radiance DN distribution (Figure 1). We averaged moment values of radiance DNs across all multispectral bands for a single observation (a given quadrat at a given recording height in each sampling event) to calculate overall distributional moments. Thus, here we define the spectral coefficient of variation as the mean coefficient of variation in the spectral radiance across raster pixels and multispectral bands for a single image. Observations were discarded if the image recording height was >8 m and replicate images were not obtained for all quadrats at all image recording heights. Therefore, the final sample size for the averaged spectral moment data was 193. In addition, to identify the spectral bands that were most sensitive to biodiversity metrics, we also tested band‐level associations, where radiance distributions were not averaged across spectral bands for the same raw data, and in this case, spectral radiance distributions of each band were related to biodiversity indices.

### 2.4 Statistical analyses

We explored the efficacy of spectral radiance distribution moments in describing in situ biodiversity indices using a Bayesian hierarchical linear regression model selection framework in the *brms* package (Bürkner, 2017; Figure 1). All variables were z‐scored (mean and variance centered on 0) for analysis to meet the distributional assumptions of linear regressions. The key response variable was the spectral coefficient of variation (Gholizadeh et al., 2019), and the key predictor variables were the in situ biodiversity indices. However, we also tested other spectral moment‐biodiversity associations, namely, the skewness of spectral radiance and biomass.

We then estimated the out‐of‐sample predictive performance of models including biodiversity indices relative to base models. For each explored pair‐wise combination of spectral distribution moment and biodiversity indices, we performed leave‐one‐out cross‐validation with the *loo* criterion and the expected log‐wise predictive density (elpd, where ∆elpd gives the change in elpd relative to another explanatory model; Vehtari et al., 2017). Base models did not include any predictor variables, including only an intercept‐only random effect for quadrat. We also investigated the performance of models including image recording height, and two‐way interaction terms between biodiversity indices and height to explore how image resolution change influenced the efficacy of spectral diversity indices.

In addition to models on averaged spectral moments, we also used band‐level moments to investigate the relationship between biodiversity indices and the spectral coefficient of variation for each individual EM band. We included univariate and two‐way interaction terms between the EM band and biodiversity indices variables. Finally, because a small number of quadrats used in the current study were also exposed to long‐term drought/irrigation/control treatments, we explored whether there were differences in average spectral moments between ambient (*n* = 25 plots), control (*n* = 2), irrigated (*n* = 5) and drought (*n* = 5) using a categorical predictor for treatment.

To account for repeated observations from the same quadrat at different heights or across sampling events, all models included an intercept‐only random effect for the quadrat $\sigma_{\text{quadrat}}$. From this random effect, we also estimated the intraclass correlation (ICC) or repeatability (R). This term indicates the proportion of quadrat‐level variance $\sigma_{\text{quadrat}}$ with respect to the population‐level variance σ (Nakagawa & Schielzeth, 2010). We used this estimate of repeatability to assess the consistency of spectral radiance distributions across observations of the same quadrat.

In analyses with average spectral distribution moments, we used weakly informed normal priors for the population‐level intercept and coefficient terms of $N\left(0,1\right)$. The $\sigma_{\text{quadrat}}$ term was fit using an exponential prior with a rate of two. For band‐level analyses (with a greater number of parameters), models were fit using $N\left(0,0.7\right)$ intercept/coefficient priors and exponential $\sigma_{\text{quadrat}}$ priors with a rate of four. Models were run across four serial chains for 2000 iterations with 1000 warmup iterations, and the model's convergence across chains was assessed by inspecting $\hat{R}$ values (Bürkner, 2017).

## 3 RESULTS

**FIGURE 2:**
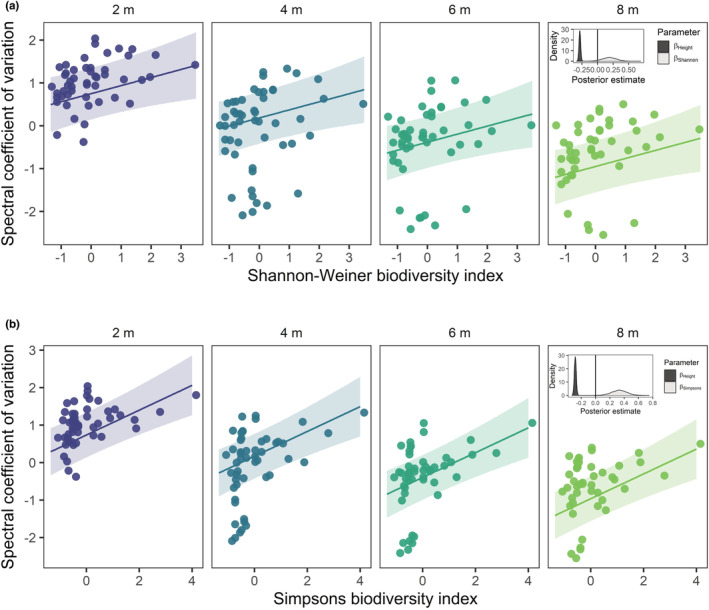
Consistent positive associations between biodiversity indices and the average spectral coefficient of variation. The positive association between (a) Shannon–Weiner biodiversity index and (b) Simpsons index, and the average spectral coefficient of variation (averaged across five spectral bands) for different image recording heights: 2, 4, 6, and 8 m (panels). Points are observations from a single quadrat at a given height. Both biodiversity indices and spectral coefficient of variation are z‐scored. Lines are the posterior prediction mean over 4000 simulations averaged over all quadrats, with the 90% credible intervals. Insets showcase density distributions of the posterior estimates for the image recording height ($\beta_{\text{height}}$) and biodiversity indices ($\beta_{\text{Shannon}}$ and $\beta_{\text{height}}$).

We found consistent positive associations between the average coefficient of variation in spectral radiance and biodiversity, namely, the Shannon–Weiner and Simpson's indices (Figure 2). The model including the Shannon–Weiner index and image recording height as univariate terms outperformed the base model, with ∆elpd=123.6 (Table S1). Increases in the Shannon–Weiner index were associated with increases in the average spectral coefficient of variation ($\beta_{\text{Shannon}}$ = 0.19 [−0.04, 0.43], 95% credible intervals; Figure 2a). Furthermore, as expected, there was a strong negative association between image recording height and the average spectral coefficient of variation ($\beta_{\text{height}}$ = −0.28 [−0.31, −0.26]; Figure 2a). This negative association suggests that the resolution of spectral diversity decreases rapidly with recording height at this spatial resolution (~30% decrease in scaled spectral variation per 1 m height increase). The positive association with the spectral coefficient of variation was stronger for the Simpson's biodiversity index (Figure 2b). Although the full model including a two‐way interaction between recording height and Simpson's index was the best predictive model, we selected the model including only univariate effects (∆elpd=125.0), because of a lack of a clear interaction effect (Table S2). Here, a similar patten with image recording height was accompanied by a stronger positive association between Simpson's index and spectral coefficient of variation ($\beta_{\text{Simpsons}}$ = 0.33 [0.12, 0.54]; Figure 2b).

Generally, we did not observe associations between the skewness and kurtosis in spectral radiance distributions and biodiversity indices (Figure S3). Furthermore, there was no clear evidence for a relationship between total above‐ground biomass and any of the spectral distribution moments (Figure S3). Specifically, although there was increased model predictive performance from models including biomass, there was no clear relationship between the skewness of spectral radiance distribution and biomass ($\beta_{\text{biomass}}$ = 0.08 [−0.19, 0.35]; Figure S3; Table S5).

**FIGURE 3:**
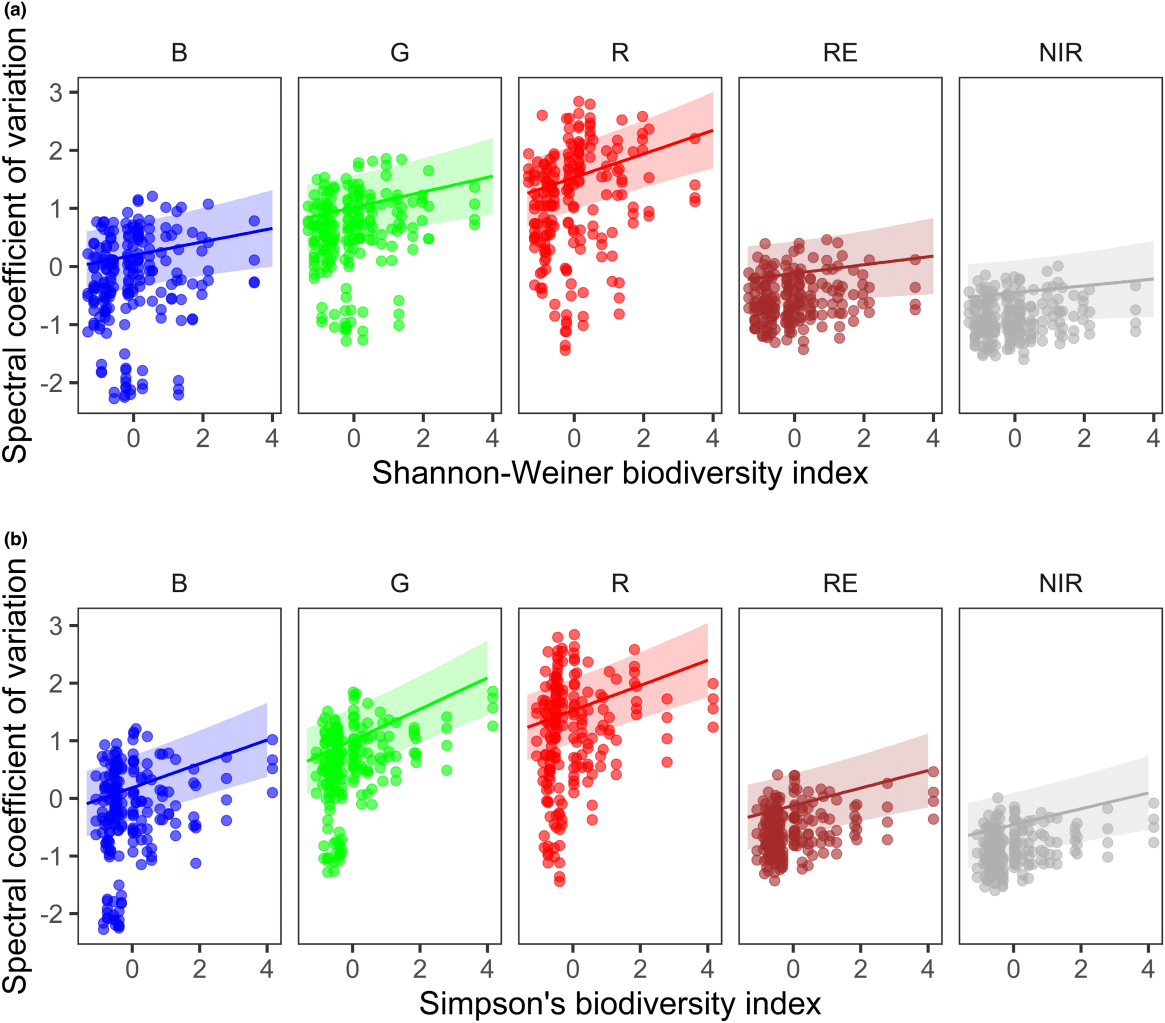
Green and red spectral bands are the most sensitive to biodiversity indices. Posterior predictions for the band‐level spectral coefficient of variation with the Shannon–Weiner (a) and Simpson's (b) biodiversity indices at an image recording height of 2 m. here, raw spectral moments were not averaged across spectral bands (as in Figure 2). Points are raw observations from a single plot, and the color denotes the spectral band (B = blue, G = green, R = red, RE = red edge, NIR = near infrared). Both biodiversity indices and spectral coefficient of variation are z‐scored. Lines are the posterior prediction mean over 4000 simulations averaged over plots, with the 90% credible intervals.

In addition to overall effects, in band‐level analyses where raw data were not averaged across bands, there was evidence for an interaction effect between the spectral band and both the Shannon–Weiner (∆elpd=772.0) and Simpson's indices (∆elpd=771.5; Tables S3 and S4, respectively). Generally, the green (560 ± 16 nm) and red (650 ± 16 nm) spectral bands displayed higher variability in the coefficient of variation across quadrats, and stronger associations with the Shannon–Weiner and Simpson's indices (Figure 3). The Red Edge (730 ± 16 nm) and Near Infrared (840 ± 16 nm) bands exhibited weaker associations with biodiversity indices (Figure 3).

When assessing the influence of treatment on spectral radiance, we also observed reductions in the average spectral coefficient of variation in both drought and procedural control quadrats in comparison to ambient or irrigated treatments (Figure S4). However, given the congruence of procedural control and drought treatments in DroughtNet, both of which are characterized by metal rainfall shelters, we conclude that the reduction in spectral coefficient of variation in drought and procedural treatments is likely a result of structural interference from the rain shelter structures (Figure S5).

Finally, we tested the consistency of the spectral coefficient of variation across observation days and heights for each quadrat in the best predictive Shannon–Weiner and Simpson's index models (Tables S1 and S2, respectively). The average coefficient of variation was highly consistent for each quadrat when images at different heights or across sampling events were compared (Figure S6). Both the Shannon–Weiner and Simpson's models with the average coefficient of variation exhibited quadrat‐level variance that exceeded the population‐level variance and a high degree of repeatability (Figure S6; 0.76 [0.65, 0.85] and 0.72 [0.60, 0.82], respectively).

## 4 DISCUSSION

Despite rapid technological advancements in image sensing over the last four decades, biodiversity monitoring is not currently able to track the full extent of human impacts on the biosphere (Wang & Gamon, 2019; Wilson, 2017). We urgently need more cost‐effective and widely available systems to monitor detailed changes in biodiversity (Cavender‐Bares et al., 2022; Turner, 2014; Turner et al., 2003). Here, using a commercially available short‐range Unmanned Aerial Vehicle (UAV, drone) at a fine spatial resolution but a coarse spectral resolution, we find a consistent association between variation in spectral radiance and species diversity in a temperate calcareous grassland. The coefficient of variation in spectral radiance was positively associated with the Shannon–Weiner and Simpsons indices, and in particular, the green and red bands of the electromagnetic (EM) spectrum were most indicative of grassland biodiversity. Our results build on extensive work in grassland ecosystems exploring the use of spectral diversity as a surrogate for biodiversity (Conti et al., 2021; Frye et al., 2021; Gholizadeh et al., 2019; Villoslada et al., 2020) and species composition (Lopatin et al., 2017). However, our research in a diverse temperate grassland community contrast with previous findings that highlighted limitations to the characterization of biodiversity using spectral imaging in species‐rich environments (Fassnacht et al., 2022; Imran et al., 2021). We highlight the importance of close‐range remote sensing for biodiversity monitoring (Turner, 2014; Turner et al., 2003; Wang & Gamon, 2019). Crucially, we demonstrate the feasibility of the spectral diversity concept in grasslands using a commercially available UAV with a coarse spectral resolution sensor, which has far‐reaching potential as a tool to explore biodiversity change at high spatio‐temporal resolution.

The key advantage of using commercially available UAV technology is its cost‐effectiveness relative to reliance on in situ monitoring or the use of long‐range or high spectral resolution sensors. In the current study, where we collected data from 37 quadrats at four different image recording heights, the total flight time was 134 min. If we reasonably allocate one researcher 60 min to do a full botanical survey (species percentage cover and biomass clip—ignoring biomass processing of 30 min per sample), the full in situ sampling time is 37 h. Thus, remote monitoring would have saved on sampling time by a factor of 16. In the current study, the reduction in sampling time is of course limited to proxies of broad biodiversity metrics. However, with increases to sensor resolution and decreasing costs, reconstructing species‐level biodiversity data with flexible remote monitoring may also be possible (Lopatin et al., 2017). Increases in cost‐effectiveness may also be increased by automated flight paths over survey locations, for which unsupervised spectral reflectance data could be collected. Furthermore, while rapid advancements have been made on spectral diversity, previous studies have utilized high‐resolution multi/hyperspectral sensors that are either immobile, destructive, or high‐cost (Frye et al., 2021; Gholizadeh et al., 2019; Imran et al., 2021; Lopatin et al., 2017). The current complete monitoring system is available for purchase for <10,000 USD, relative to >50,000 USD for many hyperspectral sensors. Interestingly, variance in the radiance of the green (560 nm) and red (650 nm) bands in the visible portion of the EM spectrum was most associated with biodiversity in this study. The red and green bands typically characterize photosynthetic pigment responses (Gamon et al., 1992). Therefore, differences between photosynthetic pigments between species may be appropriate for characterizing biodiversity in calcareous grasslands, which is possible with low‐cost visible range sensors. Nevertheless, the present study provides a low‐cost solution that successfully characterizes biodiversity in a calcareous grassland using coarse multispectral data.

The rapid increase in the availability and public use of drone technology provides an opportunity for the expansion of detailed biodiversity monitoring at flexible spatio‐temporal scales (Colomina & Molina, 2014). Integrating spatial scales has long been a central issue in remote sensing applications (Gamon et al., 2020; Turner, 2014). In the current study, images were taken with pixel resolutions between 0.1 and 0.5 cm, which lies within the range of pixel resolutions that give strong spectral diversity‐biodiversity relationships in North American prairie grasslands (Gamon et al., 2020; Wang et al., 2018). However, we still found evidence of smoothing effects (reduction in spectral diversity with coarse pixel resolutions), where the mean spectral coefficient of variation reduced between 2 and 10 m image recording height. Nevertheless, this smoothing did not influence associations with biodiversity in the current study, although this is likely due to the narrow range of pixel resolutions. While this pixel resolution is generally appropriate for grassland flora, other habitats with organisms of different sizes, functions, or differing community complexity will influence the appropriateness of the spatial resolution (Wang et al., 2018). Novel dissimilarity approaches have been applied to satellite imaging data at a range of spatial scales (Rossi et al., 2021), but ultimately there is a need for specific image sensor tools that are able to monitor spectral diversity for varied habitats. We propose that commercially available UAV technology, which can span a range of flying heights and thus spatial resolutions, can be a valuable biodiversity monitoring tool when paired with appropriate image sensors. Future application of UAV imaging to a diverse range of habitats and spatial scales is needed to fully test the utility of UAVs in biodiversity monitoring.

Temporal resolution is also a key factor when assessing spectral‐species diversity associations (Fassnacht et al., 2022). Despite the use of repeated images from the same quadrat, the data used in the current study represent a “static” measure of biodiversity at the peak of the growing season. However, grassland communities exhibit a high degree of temporal variability, particularly in response to environmental drivers (Harrison et al., 2015; Thorhallsdottir, 1990). Fassnacht et al. (2022) found that spectral diversity‐biodiversity links in plant communities were highly dependent on temporal context including phenology and seasonality. Thus, when implementing unmanned biodiversity monitoring, understanding temporal variability in spectral diversity will be critical for future research. As well as spatial integration, the solution developed by Rossi et al. (2021) successfully applied dissimilarity indices between pairs of spectral images over the same region to disentangle temporal components of community change linked to management and phenology. However, as with spatial resolution, a finer temporal resolution of data is needed to disentangle these features in plant communities (Rossi et al., 2021). Cost‐effective UAV technology has the potential to gather fine‐scale temporal data effectively.

Drone technology also has the potential to be deployed in a wide range of habitats, and to answer an array of ecological questions when combined with novel analytical tools. Indeed, there have been several recent applications of image‐sensing concepts to other habitats and in conjunction with machine learning tools to further understand community dynamics (Heim et al., 2019; Lopatin et al., 2017; Tait et al., 2019). For example, UAVs with multispectral imaging sensors have recently been applied to characterize fungal disease in lemon myrtle trees (Heim et al., 2019) and macroalgal community structure in intertidal habitats (Tait et al., 2019). The ultimate goal for biodiversity is to recreate species lists using classification algorithms. Currently, machine learning has been applied to agricultural imaging challenges (Heim et al., 2019) and in static species‐cover assessments (Lopatin et al., 2017). We argue that the applicability of cost‐effective UAV technology to biodiversity will be greatest when increased volumes of image data are combined with machine learning algorithms to identify single species or high‐resolution community dynamics. However, biodiversity metrics are not the only ecological indicators, and functional traits are also widely used as ecological indicators of environmental change (e.g., Bjorkman et al., 2018). Because spectral reflectance is related to functional form (Gamon et al., 1997), the applicability of sensing technology is not limited to biodiversity, and can also act as a proxy for functional diversity (Cavender‐Bares et al., 2022; Frye et al., 2021). In the current study site, incorporating UAV monitoring to long‐term experimental manipulations at high spatio‐temporal resolution will enable the investigation of how environmental disturbances such as drought and nutrient addition influence biodiversity, functional diversity and community structure.

## 5 CONCLUSIONS

Taking advantage of technological advancements in unmanned sensing will greatly improve the cost‐effectiveness of biodiversity monitoring. UAVs have the potential to span spatial scales, repeatedly and cheaply access a range of environments, and provide high‐resolution data on the impact of environmental change on ecosystems. Our study adds to a growing body of literature highlighting links between spectral and species diversity. Integrating these patterns at varying spatio‐temporal scales and in novel habitats will provide vital insights to aid in documenting changes in the biosphere.

## AUTHOR CONTRIBUTIONS

**John Jackson:** Conceptualization (lead); data curation (lead); formal analysis (lead); investigation (lead); methodology (lead); project administration (equal); validation (lead); visualization (lead); writing – original draft (lead); writing – review and editing (lead). **Clare S. Lawson:** Data curation (equal); investigation (equal); validation (equal); writing – review and editing (equal). **Celestine Adelmant:** Data curation (supporting); investigation (supporting); methodology (supporting); writing – review and editing (supporting). **Evie Huhtala:** Data curation (supporting); investigation (supporting); methodology (supporting); writing – review and editing (supporting). **Philip Fernandes:** Data curation (supporting); investigation (supporting); methodology (supporting); writing – review and editing (supporting). **Rose Hodgson:** Data curation (supporting); investigation (supporting); methodology (supporting). **Hannah King:** Investigation (supporting); methodology (supporting); writing – review and editing (supporting). **Lucy Williamson:** Investigation (supporting); methodology (supporting); writing – review and editing (supporting). **Kadmiel Maseyk:** Funding acquisition (supporting); methodology (supporting); project administration (equal); resources (supporting); writing – review and editing (equal). **Nick Hawes:** Conceptualization (equal); funding acquisition (equal); project administration (supporting); resources (equal); supervision (supporting); writing – review and editing (supporting). **Andrew Hector:** Conceptualization (equal); data curation (equal); funding acquisition (supporting); investigation (equal); project administration (equal); resources (equal); supervision (equal); writing – review and editing (equal). **Rob Salguero‐Gómez:** Conceptualization (equal); data curation (supporting); funding acquisition (lead); investigation (supporting); methodology (supporting); project administration (lead); resources (lead); supervision (lead); writing – original draft (equal); writing – review and editing (equal).

## CONFLICT OF INTEREST

The authors declare that there are no competing interests.

### OPEN RESEARCH BADGES

This article has earned Open Data and Open Materials badges. Data and materials are available at 10.5281/zenodo.7043832, https://github.com/jjackson‐eco/multispectral_biodiversity.

## Supporting information


Data S1:

## Data Availability

The code, data, and figures for the current study can be found in the following Zenodo archive with DOI 10.5281/zenodo.7043832, which was made from the following GitHub repository https://github.com/jjackson‐eco/multispectral_biodiversity.
